# Count on dopamine: influences of COMT polymorphisms on numerical cognition

**DOI:** 10.3389/fpsyg.2013.00531

**Published:** 2013-08-15

**Authors:** Annelise Júlio-Costa, Andressa M. Antunes, Júlia B. Lopes-Silva, Bárbara C. Moreira, Gabrielle S. Vianna, Guilherme Wood, Maria R. S. Carvalho, Vitor G. Haase

**Affiliations:** ^1^Laboratório de Neuropsicologia do Desenvolvimento, Departamento de Psicologia, Universidade Federal de Minas GeraisBelo Horizonte, Brazil; ^2^Programa de Pós-graduação em Neurociências, Instituto de Ciências Biológicas, Universidade Federal de Minas GeraisBelo Horizonte, Brazil; ^3^Faculdade de Medicina, Universidade Federal de Minas GeraisBelo Horizonte, Brazil; ^4^Programa de Pós-graduação em Genética, Instituto de Ciências Biológicas, Universidade Federal de Minas GeraisBelo Horizonte, Brazil; ^5^Department of Neuropsychology, Institute of Psychology, Karl-Franzens University of GrazGraz, Austria; ^6^Departamento de Biologia Geral, Instituto de Ciências Biológicas, Universidade Federal de Minas GeraisBelo Horizonte, Brazil

**Keywords:** catechol-O-methyltransferase, COMT, numerical cognition, non-symbolic magnitude, symbolic magnitude, dopamine

## Abstract

Catechol-O-methyltransferase (COMT) is an enzyme that is particularly important for the metabolism of dopamine. Functional polymorphisms of COMT have been implicated in working memory and numerical cognition. This is an exploratory study that aims at investigating associations between COMT polymorphisms, working memory, and numerical cognition. Elementary school children from 2th to 6th grades were divided into two groups according to their COMT val158met polymorphism [homozygous for valine allele (*n* = 61) *vs*. heterozygous plus methionine homozygous children or met+ group (*n* = 94)]. Both groups were matched for age and intelligence. Working memory was assessed through digit span and Corsi blocks. Symbolic numerical processing was assessed through transcoding and single-digit word problem tasks. Non-symbolic magnitude comparison and estimation tasks were used to assess number sense. Between-group differences were found in symbolic and non-symbolic numerical tasks, but not in working memory tasks. Children in the met+ group showed better performance in all numerical tasks while val homozygous children presented slower development of non-symbolic magnitude representations. These results suggest COMT-related dopaminergic modulation may be related not only to working memory, as found in previous studies, but also to the development of magnitude processing and magnitude representations.

## Introduction

Dopamine, a neurotransmitter of the monoaminergic catecholamine group, is engaged in several brain and cognitive functions. Traditionally, dopamine is implicated in information processing in prefrontal-related working memory tasks (Dickinson and Elvevåg, 2009; Mier et al., 2010). Among other domains, working memory is important for numerical cognition (Geary, 1993; Rotzer et al., 2009; Geary et al., 2012). For this reason, dopamine determines performance in numerical tasks always when working memory is required. Besides general cognitive factors such as working memory, specific cognitive capacities such as symbolic and non-symbolic magnitude representation also determine mathematics abilities (Mazzocco et al., 2011). Interestingly, there is compelling evidence relating basic counting processes to dopamine concentration on the prefrontal cortex (PFC; Allman et al., 2012). Could it then be that a more direct link between dopamine function and math achievement also exists? In case there is a direct connection between dopamine and arithmetic performance, which aspects of numerical processing are more influenced by variability in dopamine bioavailability? In this article, we set out to explore Catechol-O-methyltransferase (COMT) variability in the population as a clue to disentangle a hypothetical connection between dopamine bioavailability and numerical cognition.

COMT is important to the degradation of several cathecholamines, but it is especially relevant to the metabolism of dopamine, as it is responsible for degrading more than 60% of PFC dopamine (Karoum et al., 1994). Thus, COMT polymorphisms allow investigation of interactions between dopaminergic and cognitive functions. The COMT is one of the enzymes that inactivate dopamine in the synaptic cleft (Standaert and Galanter, 2009). A nucleotide substitution (G to A) at codon 158 of the COMT gene creates a functional polymorphic variation (valine158methionine, or just val158met) of this enzyme, which is especially important in the PFC (Weinshilboum et al., 1999). According to Chen et al. (2004), the enzyme met158met has a degradation rate between ⅓ and ¼ slower than the enzyme val158val. Individuals who are homozygous for the valine allele (val) have higher COMT activity and lower concentrations of extracellular dopamine. Furthermore, individuals homozygous for the methionine allele (met) tend to have lower levels of COMT activity and, consequently, higher bioavailability of dopamine in the synaptic cleft. Also according to Chen et al. (2004), the heterozygous polymorphism (val158met) has an intermediate enzyme activity.

Different patterns of information processing have been associated with the val158met COMT polymorphism (Stein et al., 2006). These differences are assumed to reflect variability in dopamine bioavailability at the synaptic cleft in the PFC. Homozygous met158met individuals tend to have what is called in the literature a more “cautious style” of information processing, with better focusing of attention as well as higher working memory capacity (“worrier strategy,” Stein et al., 2006). Otherwise, val158val homozygous individuals are prone to having a more exploratory style, which favors impulsivity and adaptation under stress conditions (“warrior strategy”). A meta-analysis of adult studies showed that there is an association between the COMT genotype and PFC activation, with a moderate to large effect size (*d* = 0.73, without evidence for publication bias; Mier et al., 2010). This study also showed that, in addition to exhibiting a lower PFC activation, the methionine allele carriers performed better in working memory tasks, while the valine allele carriers exhibited higher PFC activation levels and worse behavioral performance in these tasks. Although some studies fail to disclose differences between the COMT genotypes on working memory tasks (Mills et al., 2004; Tan et al., 2007; Blanchard et al., 2011; Wardle et al., 2013), meta-analytic evidence is compelling and less biased than the results of single studies.

Research based on child samples is scarce. Some studies support the view that met carriers are favored in working memory tasks, such as demonstrated by Diamond et al. (2004) in children aged between 6 and 14 years old. In addition, COMT has been found to modulate adolescents' behavior on two tasks of working memory (digit span and Corsi blocks), but not on a motor speed measure (Wahlstrom et al., 2007). Other evidence suggests that the association between the COMT polymorphisms and working memory may be moderated by age. Thus, analyzing individuals aged between 6 and 20 years old, Dumontheil et al. (2011) found that polymorphic-related working memory differences were present only in children older than 10 years.

The relevance of working memory for mathematical learning is well-established, regarding both typical and atypical development (Raghubar et al., 2010). As an example, in previous research (Costa et al., 2011), we found that working memory effects on simple word problems were attenuated by including finger gnosia as covariate in the analyses. As finger discrimination abilities presumably enable finger counting, this result may be interpreted in terms of the offloading mechanisms proposed by Alibali and DiRusso (1999, see also Costa et al., 2011). The executive component of working memory is more strongly implicated in math performance than storing mechanisms (Raghubar et al., 2010).

Another important contribution of working memory is related to number transcoding (changing the type of numerical representation, for example: “4” −> “four”). Children with higher counting span show superior performance in a numerical transcoding task compared to those with lower span (Camos, 2008). In that study, working memory level was also correlated with the complexity of the items to be transcoded. Another study examining the transcoding abilities of 7-years-old children suggested that visuospatial working memory predicts the performance in transcoding tasks in the context of German language, in which there is decade-unit inversion in two-digit numbers (Zuber et al., 2009). According to Barrouillet et al. (2004), working memory resources are increasingly required for transcoding complex numbers. Evidence also indicates that transcoding may be performed as a purely algorithmic procedure, not requiring access to semantic representations of magnitude (Deloche and Seron, 1982a,b; Barrouillet et al., 2004).

The connection between the val158met COMT polymorphism, working memory, and mathematical achievement was explored in a study of MRI-related events by Tan et al. (2007). Individuals who carried the valine allele showed higher activation levels of the dorsolateral PFC in comparison to individuals with the met/met COMT genotype even in the absence of differences in behavioral performance. COMT activity levels were positively correlated with the maintenance and processing of information in working memory during mathematical tasks, but not with retrieval operations from long-term memory. These pieces of evidence indicate that individuals with lower dopamine levels (met/met homozygous) can perform the same operations presumably recruiting less working memory resources.

Besides working memory capacity, several other abilities and cognitive representations such as magnitude representation are recruited in number processing and calculation (Prado et al., 2011). The non-symbolic magnitude representation is the capacity to analogically, intuitively, and automatically discriminate, estimate, and compare magnitudes. The non-symbolic magnitude representation can be observed already in infants (Xu et al., 2005) and is very basic (Dehaene, 1992). Operations with non-symbolic magnitudes are executed in an approximate manner. Accuracy of the magnitude representation may be assessed by the Weber fraction (the minimal proportional numerical difference that may be discriminated between two sets of objects), or by the coefficient of variation in estimation of the numerosity of sets (Gilmore et al., 2011). This inverse association between numerical distance and reaction times and error rates is known as the distance effect, and it represents an instantiation of the Weber law (Dehaene, 1989; Dehaene et al., 1990).

A direct link between dopaminergic modulation and magnitude estimation is postulated in the accumulator model (Meck and Church, 1983). This model comes from temporal information processing research (Gibbon, 1977), and was successfully generalized to numerical cognition (Meck and Church, 1983; Dormal and Pesenti, 2012). It proposes the existence of a pacemaker that emits pulses which are gated into an accumulator. The pulse transmission is regulated by two different modes: a counter, when operating in the event mode (numerical estimation) or a clock, when operating in the run mode (time estimation). The accumulator value can be held in working memory and compared to a previous accumulator value stored in reference memory. A decision process determines the appropriate response by discriminating which of the two values is the larger one (Meck and Church, 1983; Allman et al., 2012). Meck and Church (1983) obtained evidence in rodents that learning of numerosity and time estimation have similar psychophysical functions, interfere one with another and are similarly affected by pharmacological manipulations. These results suggest that a common magnitude processing mechanism underlies time and numerosity estimations (Walsh, 2003; Dormal and Pesenti, 2012).

Meck (2006) showed, in animals that lesions in frontal areas led to selective reduction of the modulatory control of the clock speed, interfering with estimation of duration. Results also indicated that this speed reduction was mediated by dopamine receptors. More precisely, dopamine agonists have a dose-dependent influence on magnitude estimation (Meck and Church, 1987; Lustig and Meck, 2005; Cheng et al., 2008). It was verified that the administration of methamphetamine (a dopamine agonist) produced an overestimation in time and numerical responses of rats. In other words, higher dopamine bioavailability (provocated by the methamphetamine) was associated with a distorted perception of time and numbers in a non-human sample (Meck and Church, 1983). Balci et al. (2013) showed that the COMT polymorphisms that lead to higher prefrontal dopamine resulted in weaker manifestation of memory variability in timed behavior. More specifically, the association between COMT and time comparison has been studied by Wiener et al. (2011), who showed that the COMT polymorphism influences the perception of time magnitude, with individuals with at least one methionine allele presenting a better performance (smaller coefficients of variation). Thus, it is plausible to establish a relationship between COMT and the mechanisms of magnitude representation (Coull et al., 2011).

The role of working memory and magnitude representations in arithmetic learning suggests, therefore that an important source of interindividual variation may be related to COMT polymorphisms. They can have an impact on dopaminergic functions related to working memory and also possibly to magnitude representations. This exploratory study aims at: (1) investigating the influence of dopamine bioavailability, operationalized by means of val158met polymorphism of the COMT enzyme, on the performance of working memory tasks; (2) assessing possible differences between COMT polymorphic groups on numerical tasks; and (3) exploring the interplay between dopamine bioavailability, working memory, and symbolic and non-symbolic numerical tasks. We hypothesize that met carriers will have better performance on numerical tasks requiring working memory abilities due to their higher levels of dopamine in the synaptic cleft. The relevance of COMT polymorphisms for non-symbolic representations is still speculative. However, based on the previously presented evidence, one can argue that met carriers should also have a better performance on basic non-symbolic numerical tasks. Therefore, this study also aims at investigating whether there are group differences in non-symbolic tasks between the different COMT allele combinations. If the effects of COMT on number processing are not completely mediated by working memory capacity, between group differences should remain present in numerical tasks even after removing the impact of working memory capacity.

## Materials and methods

### Participants

Two hundred and nineteen school-aged children were recruited from public and private schools from Belo Horizonte, Brazil. This study was approved by the local research ethics committee. Participation occurred only after informed consent was obtained in written form from parents or the legal representatives, and orally from children. All children had normal intelligence and did not present any major psychiatric or neurological illnesses. Data from 64 children were not included in the analyses, because they did not complete one of the tests (*n* = 33), had a poor *R*^2^ on the fitting procedure to calculate their internal Weber fraction on the non-symbolic comparison task (*R*^2^ < 0.2; *n* = 13) and/or were outliers and extreme cases on any of the instruments used (*n* = 18), which might suggest they were not able to solve the task or were not paying adequate attention to it.

The final sample was constituted by 155 children with ages ranging from 8 to 12 years. They were attending elementary school grades ranging from 1st to 6th. Sixty-one children were homozygous for the valine allele (val/val), 18 children were homozygous for the methionine allele (met/met), and 76 children were heterozygous (val/met). No deviations from Hardy–Weinberg Equilibrium were observed in our study (*x*^2^ = 0.001; *p* > 0.05). Due to the small number of homozygous subjects for the methionine allele, we divided the sample into two broader groups: (a) children homozygous for the valine allele (thereafter *val/val, n* = 61); and, (b) children with at least one methionine allele (thereafter *met*+, *n* = 94). These two groups were matched by age, grade, sex and intelligence (all *p* > 0.05). Only 7% of the final sample was left-handed. Demographic data are described in Table 1.

**Table 1:** **Descriptive data of the sample distribution according to COMT genotype**.

	**val/val**	**met+**	**χ**^**2**^	***df***	***P***
*N*	61	94	–	–	–
Sex (%female)	59.00	59.60	<0.01	1	0.95
	**Mean (SD)**	**Mean (SD)**	***t***	***df***	***P***
Age (years)	9.61 (1.20)	9.81 (1.10)	−1.08	153	0.28
Raven (z-score)	0.43 (0.75)	0.52 (0.70)	−0.82	153	0.41

### Genetic analysis

Ten milliliters of peripheral blood samples were collected and DNA was extracted with proteinase K digestion followed by a saline precipitation protocol (Miller et al., 1988). DNA samples were quantified using an optical absorbance spectrophotometer. COMT val158met polymorphism was genotyped with tetra-primer amplification refractory mutation system-polymerase chain reaction (ARMS-PCR), as described in the literature (Ruiz-Sanz et al., 2007). ARMS-PCR reactions contained 200–400 ng of genomic DNA, 0.1 μM of P1 primer, 0.05 μM of P2 primer, 0.75 μM of P3 primer, 0.1 μM of P4 primer, 0.5 μM of dNTPs, 5% DMSO, and 0.1 U *Taq* DNA polymerase in a 20 μL final volume. The PCR cycling program included an initial denaturation step at 94°C for 4 min, followed by 30 cycles of denaturation at 94°C for 30 s, annealing at 58°C for 30 s, and extension at 72°C for 45 s, which was followed by last extension step extension at 72°C for 5 min. ARMS-PCR products were resolved electrophoretically on 6% polyacrylamide gels, stained with silver nitrate. This ARMS-PCR amplification system may produce three amplicons. A 626 bp amplicon is produced by amplification of P1 and P2 primer pair, functions as an intratube PCR control, and is expected to amplify in every sample containing human DNA. A 222 bp amplicon is produced by the amplification with P1 and P4 primer pair and corresponds the met allele. A 451 bp amplicon is produced by the amplification with P2 and P3 primer pair corresponds to the val allele. Approximately, 20% of the amplification reactions were confirmed by restriction with the enzyme *Hsp*92II.

### Procedure

The data collection occurred in the children's schools and was divided in three distinct phases. The first phase was conducted in group. Children were tested using an intelligence test (Raven's Colored Progressive Matrices) and the Arabic number writing task. Afterwards, parents or the legal representatives were invited to their children's schools. In this occasion, biological sample collection (peripheral blood) was performed. The third phase was the individual neuropsychological assessment. Children were tested in a quiet room in their own schools using paper and pencil neuropsychological tasks, the only exceptions being the non-symbolic numerical tasks, which were presented in a 15.4 inches laptop.

### Instruments of neuropsychological assessment

#### Raven's colored progressive matrices

General intelligence was assessed with the age-appropriate Brazilian version of Raven's Colored Matrices (Angelini et al., 1999). The z-scores were calculated based on the manual's norms.

#### Digit span

Verbal short-term and working memory were assessed with the Brazilian WISC-III Digits subtest (Figueiredo, 2002). Performance in the forward order was considered a measure of phonological short-term memory and the backward order was used to assess verbal working memory (Figueiredo and Nascimento, 2007). We evaluated the total score (correct trials × span) in the forward and the backward orders (Kessels et al., 2000).

#### Corsi blocks

This test is a measure of the visuospatial component of short-term and working memory. It is constituted by a set of nine blocks which are tapped, in a certain sequence, by the examiner and this child must repeat the same sequence. The test starts with sequences of two blocks and can reach a maximum of nine blocks. We used the forward and backward Corsi span tasks according to Kessels et al. (2000). In the forward condition, the child is instructed to tap the blocks on the same order as the examiner, in the backward condition, in the inverse order. We evaluated span in each condition.

#### Arithmetic word problems

Twelve arithmetical word problems were presented to the child on a sheet of paper while the examiner read them aloud simultaneously to avoid reading proficiency bias. There were six addition and six subtraction items, all of them with single-digit operands and results ranging from 2 to 9 (e.g., “*Annelise has* 9 *cents. She gives* 3 *to Andressa. How many cents does Annelise have now?*”). The child had to solve the problems mentally and write the answer down in the Arabic format as quickly as possible. Cronbach's α of this task was 0.83 (Costa et al., 2011). We computed the total correct response in the task.

#### Arabic number writing task (verbal to arabic transcoding task)

This is a symbolic task on which children were instructed to write the Arabic form of dictated numbers. This task is constituted by 40 items, up to 4 digits (3 one-digit numbers, 9 two-digit numbers, 10 three-digit numbers, and 18 four-digit numbers). The percentage of the correct responses in the task was computed. The internal consistency of this task is 0.96 (KR-20) (Lopes-Silva, 2013).

#### Arabic number reading task (arabic to verbal transcoding task)

Twenty eight Arabic numbers printed in a booklet were presented one at a time, to the children, who were instructed to read them aloud. The item set consists of numbers up to 4 digits (3 one-digit numbers, 9 two-digit numbers, 8 three-digit numbers, and 8 four-digit numbers). The total correct response was computed. The internal consistency of the task was 0.90 (KR-20 formula) (Lopes-Silva, 2013).

#### Non-symbolic magnitude estimation task

**Figure 1:**
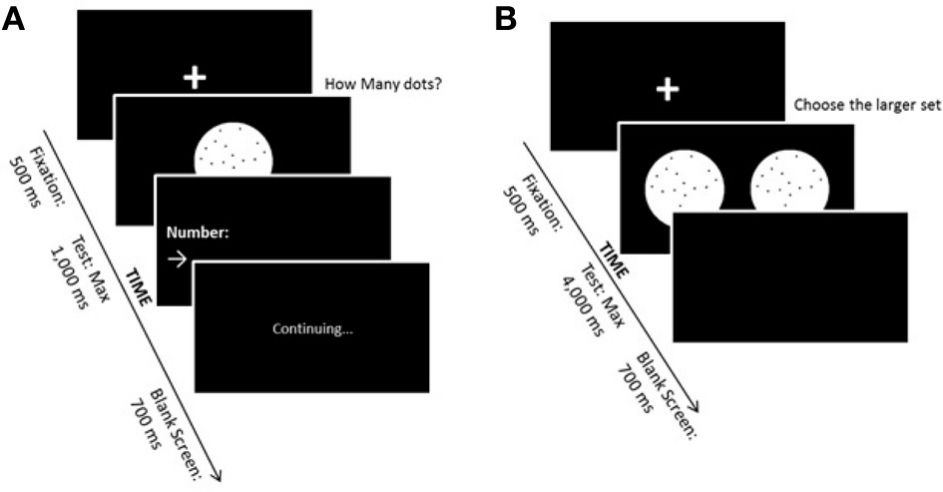
**(A)** Non-symbolic magnitude estimation task. **(B)** Non-symbolic magnitude comparison task.

In the non-symbolic magnitude estimation task, participants were asked to estimate with a verbal response the quantity of dots presented on the computer screen. Black dots were presented on a white circle over a black background. Numerosities were 1, 2, 3, 4, 5, 10, 16, 24, 32, 48, 56, or 64 dots. Each numerosity was presented 5 times, each time in a different configuration and in such a way that the same numerosity never appeared in consecutive trials. The task comprised 60 testing trials. The maximum stimulus presentation time was 1000 ms, fast enough to avoid counting. Inter-trial interval was 700 ms. As soon as the child responded, the examiner, who was sitting next to the child, pressed the spacebar on the keyboard and typed the child's answer. Between each trial, a fixation point appeared on the screen for 500 ms—a cross, printed in white, with 3 cm in each line (see Figure 1A). As a measure of non-symbolic magnitude representation acuity, we calculated the mean coefficient of variation (estimation cv mean) of the numbers ranging from 10 to 64 of the responses for each child (Pinheiro-Chagas, 2012). Numbers between 1 and 5 were not included in the analyses because they are on the subitizing range, which require an exact access to non-symbolic magnitude and it is not on the scope of the present work.

#### Non-symbolic magnitude comparison task

In the non-symbolic magnitude comparison task, the participants were instructed to compare two simultaneously presented sets of dots, indicating which one contained the larger number. Black dots were presented on a white circle over a black background. On each trial, one of the two white circles contained 32 dots (reference numerosity) and the other one contained 20, 23, 26, 29, 35, 38, 41, or 44 dots. Each magnitude of dot sets was presented eight times. The task comprised 8 learning trials and 64 experimental trials. Perceptual variables were varied such that in half of the trials individual dot size was held constant, while in the other half the size of the area occupied by the dots was held constant (see exact procedure descriptions in Dehaene et al., 2005). Maximum stimulus presentation time was 4000 ms, and inter-trial interval was 700 ms. before each trial, a fixation point appeared on the screen—a cross, printed in white, with 30 mm in each line. If the child judged that the right circle presented more dots, a predefined key localized in the right side of the keyboard should be pressed with the right hand. On the contrary, if the child judged that the left circle contained more dots, then, a predefined key on the left side had to be pressed with the left hand (Costa et al., 2011; see Figure 1B). As a measure of the acuity of the ANS, the *internal Weber fraction* (thereafter *w*) was calculated for each child individually. The proportion of responses in which the target was considered to be larger than the reference was determined for each numerical ratio tested. The proportion of these responses obtained for each ration were plotted and a Log-Gaussian predictor was estimated. The *R*^2^ obtained for each child was used as a reference for the individual goodness-of-fit. Children with *R*^2^ < 0.2 were excluded from all further analyses. The slope of the Log-Gaussian corresponds to *w* (Dehaene, 2007), and describes the amount by which one would need to change a particular target in order for a given participant to correctly detect that it is larger than the reference on 75% of the trials (Piazza and Dehaene, 2004).

## Results

Analyses of variance (ANOVA) were run to identify significant group differences between met+ and val/val groups regarding working memory and numerical abilities. To investigate the extent to which the Weber fraction (*w*) and the mean coefficient of variation from the non-symbolic magnitude estimation task (cv mean) contributed to these group differences, ANCOVA models including these two variables as covariates were calculated. Analyses in which these non-symbolic magnitude processing tasks removed group differences were interpreted as indicative of a specific influence of non-symbolic magnitude representations on other numerical and arithmetic processes.

Even though there was no statistical difference between groups regarding age (*p* = 0.28), after calculating the effect size of this difference (*d* = 0.18) we included age as a covariate in all further analyses. Generally, all results remained qualitatively unchanged, independently of age being statistically controlled or not. Therefore, we decided to focus only on the one-factor ANOVA. We also examined the correlations between all numerical tasks for each of the allelic groups separately. We considered *p*-values below 0.05 as being significant.

### COMT polymorphisms and working memory

No group difference between val/val and met+ groups was observed in working memory tasks (*p* ≥ 0.15). We compared the groups with a series of ANOVAs on Corsi Blocks and Digits Span and all *F* tests were lower than 1.00 [with the exception of Digit Span forward, on which *F*_(153)_ = 2.12] and all the effect sizes were significantly small (η^2^ ≤ 0.01).

### COMT polymorphisms and numerical and arithmetic tasks

Our second main goal was to investigate if there were any group differences in numerical tasks. Results of group comparisons are depicted in Table 2.

**Table 2:** **Analysis of variance (ANOVA) and covariance (ANCOVA) of the numerical tasks**.

**Tasks**	**val/val (*n* = 61)**	**met+ (*n* = 94)**	**ANOVA**			**ANCOVA (Covariate: Weber fraction)**		
	**Mean (SD)**	**Mean (SD)**	***F***	***p***	**η**^**2**^	***F***	***p***	**η**^**2**^
Arithmetic word problems	9.15 (2.57)	9.91 (1.98)	4.38	0.04	0.03	2.97	0.09	0.02
Arabic number reading task	25.97 (3.15)	27.15 (1.75)	8.98	<0.01	0.06	6.94	0.01	0.04
Arabic number writing task	0.85 (0.22)	0.95 (0.08)	15.64	<0.01	0.09	12.11	<0.01	0.07
Non-symbolic magnitude comparison task (*w*)	0.31 (0.16)	0.26 (0.10)	6.13	0.01	0.04	–	–	–
Non-symbolic magnitude estimation task (cv mean)	0.19 (0.07)	0.17 (0.06)	4.65	0.03	0.03	1.96	0.16	0.01

Statistically significant differences between val/val and met+ were found in all numerical tasks. Children in the met+ group presented superior performance in all numerical and arithmetical tasks (Table 2). After controlling for the impact of *w* in ANCOVA models, all comparisons remained significant, with the exception of arithmetic word problems and estimation cv mean (Table 2). Although not significant, it is important to note that the effect sizes associated with these two comparisons were moderate or small and that after removing the effect of the non-symbolic magnitude estimation (cv mean) the effect sizes remained roughly unchanged.

Moreover, the between-group differences in arithmetic word problems and *w* disappeared [*F*_(152)_ = 2.50, *p* = 0.12, η^2^ = 0.02; *F*_(152)_ = 3.41, *p* = 0.07, η^2^ = 0.02, respectively] when cv mean was the covariate. One can infer that both *w* and cv mean are functionally associated: the difference on *w* disappears when cv mean is used as a covariate and vice versa.

Finally, group differences in the Arabic number reading and Arabic number writing tasks also remained significant [*F*_(152)_ = 6.13, *p* = 0.01, η^2^ = 0.04; *F*_(152)_ = 11.45, *p* ≤ 0.001, η^2^ = 0.07, respectively] when cv mean was the covariate. This suggests that differences in transcoding skills between val/val and met+ cannot be attributed to the effect of non-symbolic magnitude representations.

To further explore the association between COMT polymorphisms, working memory and numerical achievement, we investigated the correlation between these variables in each group separately. The section above the diagonal refers to the met+ group (gray background) whereas the one below the diagonal concerns the correlation values of the val/val group (Table 3). Significant correlations were found in both groups. Arabic number reading, Arabic number writing and arithmetic word problems correlated with each other (Table 3). Moreover, digit span backward correlated with Arabic number reading task, Arabic number writing task and non-symbolic magnitude estimation task. Corsi blocks backward presented significant correlations to Arabic number writing task. Regarding the non-symbolic tasks, the magnitude estimation task correlated with the magnitude comparison task and with arithmetic word problems.

**Table 3:**
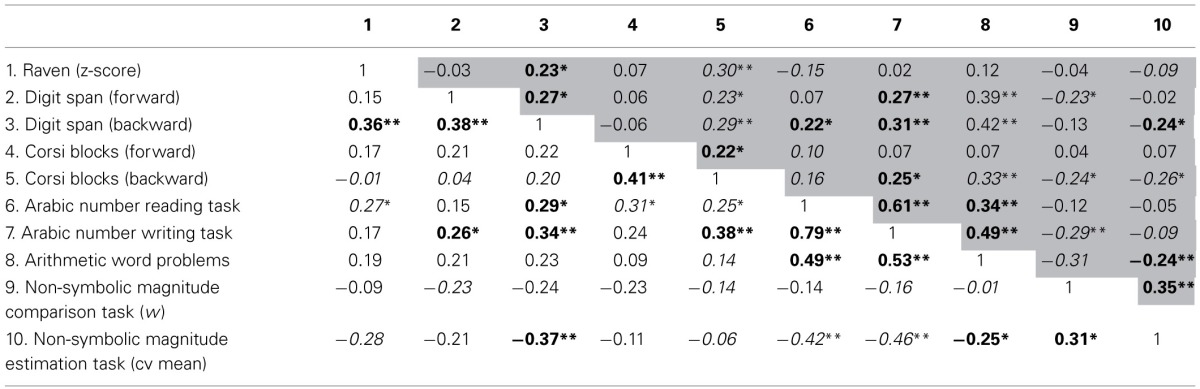
**Correlations between the neuropsychological measures of met group in the upper diagonal (gray back ground) and val/val group in the lower diagonal (white background)**.

^*^p < 0.05; ^**^p < 0.01. Correlations shown in italics were significant in the group being analyzed, but not in the other one, while correlations in bold were significant in both groups.

Similar correlation patterns were observed in the val/val and met+ groups. At first, the correlation patterns found in met carriers were analyzed. Inspection of Table 3 (upper diagonal) reveals that digit span backward did not correlate with the non-symbolic magnitude comparison task. All of numerical tasks correlated with each other, with the exception of Arabic number reading that did not correlate to w nor to estimation cv mean and the Arabic number writing task did not correlate to estimation cv mean. Surprisingly, there was a significant correlation between Arabic number writing task and *w*.

Children with the val/val genotype presented a different pattern of correlations (lower diagonal of Table 3). Arabic number reading, Arabic number writing and arithmetic word problems tasks presented significant correlations with non-symbolic magnitude estimation task, but did not correlate with the non-symbolic magnitude comparison task. All of these symbolic numerical tasks also correlated with digit span and Corsi blocks (backward orders), with except for the arithmetic word problems.

In summary, in both groups, Arabic number reading, Arabic number writing, and arithmetic word problems that rely more strongly on working memory, presented significant correlations with digit span and Corsi blocks backward. In general, both val/val and met+ groups presented a very distinctive pattern of correlations and only in the met+ group all of these numerical tasks were correlated to the non-symbolic ones.

## Discussion

In the present study the association between COMT genotypes in val158met polymorphism and numerical cognition was examined in school-aged children. Our results can be summarize as follows: (1) Children with at least one met allele (met+) showed better performance in numerical tasks when compared to children with val/val genotype. Numerical tasks encompassed simple operations (arithmetical word problems), transcoding (Arabic number reading and Arabic number writing tasks) and non-symbolic magnitude processing. Effect sizes of these difference were moderate or small, but these results were in accordance to what was expected, since the groups were stratified using a single gene as criterion; (2) when Weber fraction (*w*) was entered as a covariate in the group comparison analyses, differences on simple arithmetic word problems and on the coefficient of variation in non-symbolic estimation were no longer significant (corresponding results were also obtained when the influence of the coefficient of variation in non-symbolic estimation was removed); and (3) contrary to expectations, groups performed equally in the working memory tasks. In the following, these results will be discussed in further detail.

### COMT and non-symbolic numerical processing

Our most important result is the difference between COMT val/val and met+ groups regarding both numerical non-symbolic measures (non-symbolic magnitude comparison task and non-symbolic magnitude estimation task). This suggests a direct link between dopamine regulation and magnitude processing mechanisms that is independent from working memory capacity. Results are better contextualized in terms of the accumulator model (Meck and Church, 1983). The accumulator model assumes that dopamine modulates at least two mechanisms involved in non-symbolic number processing: working memory and clock speed (for run mode) or counter (for event mode). Research has traditionally focused on the role of val158met COMT polymorphism in working memory variability (Mier et al., 2010). For the first time, we obtained evidence that COMT-related variability, probably related to dopaminergic mechanisms, may be more directly involved with magnitude processing. Variations in dopamine bioavailability alter the clock speed, and could affect the precision with which magnitude estimations and comparisons are performed. The present results are in line with those reported by Wiener et al. (2011), who found that individuals homozygous for valine allele presented higher coefficients of variation in a time comparison task (supra-second) compared to individuals with at least one methionine allele. The present results also indicate that val homozygous presented poorer acuity in the non-symbolic magnitude comparison task, as shown by their larger Weber fraction values. Besides that, mathematical learning disabilities can be thought as a model to this relationship between time and magnitude. The deficit that these children show in non-symbolic number tasks has already been well-described (Piazza et al., 2010; Costa et al., 2011; Mazzocco et al., 2011; Mejias et al., 2012). Furthermore, a recent paper has shown that children with mathematical learning disabilities also present poorer temporal estimation when compared to typical peers (Hurks and van Loosbroek, 2012). Therefore, both time and quantity modalities seem to be associated to specific COMT polymorphisms, as predicted by the accumulator model (Meck and Church, 1983).

The present results also suggest that children in the met+ group had a more accurate non-symbolic magnitude representations. Val/val carriers had higher Weber fraction values which means that they are less able to discriminate accurately between magnitudes. In a similar way, children with learning disability also showed higher Weber fraction values (Piazza et al., 2010; Costa et al., 2011; Mazzocco et al., 2011). In contrast, better performance on non-symbolic numerical tasks has been associated with better performance in mathematical tasks in previous studies (Halberda et al., 2008). We found that Weber fraction has an impact in arithmetic word problems but not in the transcoding tasks (Arabic number reading task and Arabic number writing task): the more accurate in their non-symbolic skills the subject is, the better is the performance on the arithmetic word problems. These results are in line with the Barrouillet et al. (2004) that claims that transcoding abilities are asemantic, which means they do not require access to magnitude representations. However, this does not happen with the arithmetic word problems, which comprise only single-digit numbers and probably automatically access the magnitudes they represent. Available evidence shows that in early stages of development, when they are learning to perform single digit operations, children extensively activate prefrontal and intraparietal areas. As proficiency is acquired and operations are progressively automatically solved as arithmetic facts, the activation focus shifts from the prefrontal and intraparietal sulcus to the angular gyrus (Rivera et al., 2005). Intraparietal activation levels during single-digit arithmetic are also predictive of math performance in high school students (Price et al., 2013).

The similar results obtained when we controlled for the influence of both non-symbolic magnitude comparison and estimation suggest that these two skills interchangeably influence performance on arithmetic problems, but not on transcoding tasks. This indicates that these two measures tap a common non-symbolic (analogic) construct. However, as correlations between Weber fraction and the coefficient of variation in estimation were significant but of low magnitude (Table 3), other sources of variance may influence performance in these tasks. Although both tasks are used to access non-symbolic magnitudes, they may also recruit other cognitive components (Gilmore et al., 2011). One difference could be related to the connection to symbolic representations, more salient in the estimation task. In the context of developmental dyscalculia, Rousselle and Noël (2007; Noël and Rousselle, 2011) have suggested a distinction between deficits in non-symbolic representations and deficits in symbolic to non-symbolic access or mapping mechanisms. Performance in these two kinds of tasks is, for example, dissociable in children with math learning disability (see also Mejias et al., 2012). Some authors claim that children with developmental dyscalculia experience more difficulties in the symbolic than in the non-symbolic tasks (Rousselle and Noël, 2007; Mejias et al., 2012). In the case of our results, this could mean that val-val individuals' performance in the estimation task improves comparatively less in comparison to met+ individuals because it is a more complex task due to its symbolic requirements.

### COMT and working memory

Although some studies describe differences in working memory tasks between groups of COMT polymorphisms (Goldberg et al., 2003; Mattay et al., 2003), these results are not consistently reported in the literature (Tan et al., 2007; Blanchard et al., 2011; Wardle et al., 2013), especially for children (Diamond et al., 2004; Mills et al., 2004; Wahlstrom et al., 2007). Distinct arguments may help to explain why working memory performance did not differ across COMT polymorphic groups. The first one regards single nucleotide polymorphism effects and the different approaches the authors organize genetic data (see review in Mier et al., 2010). Some studies compare all three genotypes (val/val, val/met, and met/met) and other consider a genetic dominance as we did in the present study (val/val *vs*. met carriers or met/met *vs*. val carriers). Secondly, tasks vary from one study to the other. In our study we use digit span and Corsi blocks, but most of the previous research use the n-back paradigm that demands more “storage” capacity in contrast of the “manipulation” capacity required by our tasks. Bruder et al. (2005) proposed that these two kinds of abilities may not involve the same circuitry in PFC. As suggested by Wardle et al. (2013), some working memory tasks may be more sensitive to COMT effects than other ones. Thirdly, the study that found differences between groups using the same tasks as in our study investigated an adolescent sample (age ranging between 9 and 17) (Wahlstrom et al., 2007) and just ¼ of our sample was older than 10 years old. In this way, the results are not easily comparable. Moreover, Dumontheil et al. (2011) showed that the difference favoring met allele emerges only after 10 years of age, what can easily be conciliated with our results. At last, also consistent with our results is the finding reported in many studies, which were not able to observe behavioral effects in working memory tasks, but obtained significant differences in brain activation patterns of individuals with COMT polymorphisms (Meyer-Lindenberg et al., 2006; Caldú et al., 2007; Tan et al., 2007).

### The interplay of COMT, working memory, and symbolic numerical processing

In the present study, differences between polymorphisms arose in transcoding tasks, which knowingly require working memory resources. It is one of the cognitive mechanisms most consistently associated with Arabic number writing (Barrouillet et al., 2004; Camos, 2008). According to Barrouillet et al. (2004), verbal working memory plays a central role in the management of the slots to be filled during the transcoding procedure and this relationship is even more pronounced in more complex numbers (such as 4 digit-numbers with an internal zero, e.g., 6047). Camos (2008) also claims that working memory load is crucially involved in the acquisition of more complex transcoding rules. In her study, it was found that children with higher working memory span presented less transcoding errors, especially the ones related to the knowledgment of place-value concept. Even though we did not find group differences in our working memory tasks, one can assume that underlying differences on this skill between COMT polymorphisms might play a role in the variability on the transcoding tasks. One possible explanation is that some previous studies that found association between working memory and transcoding skills used different measures, such as dual tasks (Camos, 2008), while our measures of working memory are associated with storage and manipulation of verbal as well as spatial information. Zuber et al. (2009) found an association between Corsi blocks and number writing performance. That study was conducted in German speaking children and thus this association is possibly due to the spatial demands related to the inversion rule in German language.

In the arithmetic word problems task, we also found differences between groups. Methionine carriers children had a better performance in the arithmetic word problem task. As we did not find polymorphic group differences regarding working memory tasks, the group effect on arithmetic word problems could be explained in terms of differing activation patterns of non-symbolic magnitude representations as discussed above.

In conclusion, these findings suggest that dopaminergic mechanisms may be implicated in numerical magnitude processing. Significant differences between children with val/val vs. met+ genotypes were also detected on aspects of arithmetic achievement that demand more non-symbolic magnitude processing, such as single-digit arithmetic word problems. Our results suggest that COMT-related dopaminergic mechanisms are implicated not only on working memory-related arithmetic performance, as previously shown (Tan et al., 2007), but also on non-symbolic magnitude processing. Further investigation is necessary to clarify the association between COMT polymorphisms, working memory, and numerical cognition.

### Conflict of interest statement

The authors declare that the research was conducted in the absence of any commercial or financial relationships that could be construed as a potential conflict of interest.

## References

[B1] Alibali M. W. DiRusso A. A. (1999). The function of gesture in learning to count: more than keeping track. Cogn. Dev. 14, 37–56 10.1016/S0885-2014(99)80017-3

[B2] Allman M. J. Pelphrey K. A. Meck W. H. (2012). Developmental neuroscience of time and number: implications for autism and other neurodevelopmental disabilities. Front. Integr. Neurosci. 6:7 10.3389/fnint.2012.00007 22408612 PMC3294544

[B3] Angelini A. L. Alves I. C. B. Custódio E. M. Duarte W. F. Duarte J. L. M. (1999). Matrizes Progressivas Coloridas de Raven: Escala Especial. Manual. São Paulo: CETEPP

[B4] Balci F. Wiener M. Cavdaroğlu B. Coslett B. H. (2013). Epistasis effects of dopamine genes on interval timing and reward magnitude in humans. Neuropsychologia 51, 293–308 10.1016/j.neuropsychologia.2012.08.002 22903038

[B5] Barrouillet P. Camos V. Perruchet P. Seron X. (2004). ADAPT: a developmental, asemantic, and procedural model for transcoding from verbal to Arabic numerals. Psychol. Ver. 111, 368–394 15065914 10.1037/0033-295X.111.2.368

[B6] Blanchard M. M. Chamberlain S. R. Roiser J. Robbins T. W. Müller U. (2011). Effects of two dopamine-modulating genes (DAT1 9/10 and COMT Val/Met) on n-back working memory performance in healthy volunteers. Psychol. Med. 41, 611–618 10.1017/S003329171000098X 21272388

[B7] Bruder G. E. Keilp J. G. Xu H. Shikhman M. Schori E. Gorman J. M. (2005). Catechol-O-methyltransferase (COMT) genotypes and working memory: associations with differing cognitive operations. Biol. Psychiatry 58, 901–907 10.1016/j.biopsych.2005.05.010 16043133

[B8] Caldú X. Vendrell P. Bartrés-Faz D. Clemente I. Bargalló N. Jurado M. A. (2007). Impact of the COMT Val108/158 Met and DAT genotypes on prefrontal function in healthy subjects. Neuroimage 37, 1437–1444 10.1016/j.neuroimage.2007.06.021 17689985

[B9] Camos V. (2008). Low working memory capacity impedes both efficiency and learning of number transcoding in children. J. Exp. Child Psychol. 99, 37–57 10.1016/j.jecp.2007.06.006 17854821

[B10] Chen J. Lipska B. K. Halim N. Ma Q. D. Matsumoto M. Melhem S. (2004). Functional analysis of genetic variation in catechol-o-methyltransferase (comt): effects on mrna, protein, and enzyme activity in postmortem human brain. Am. J. Hum. Genet. 75, 807–821 10.1086/425589 15457404 PMC1182110

[B11] Cheng R. K. MacDonald C. J. Williams C. L. Meck W. (2008). Prenatal choline supplementation alters the timing, emotion, and memory performance (TEMP) of adult male and female rats as indexed by differential reinforcement of low-rate schedule behavior. Learn. Mem. 15, 153–162 10.1101/lm.729408 18323570 PMC2275657

[B12] Costa A. J. Silva J. B. L. Chagas P. P. Krinzinger H. Lonneman J. Willmes K. (2011). A hand full of numbers: a role for offloading in arithmetics learning? Front. Psychol. 2:368 10.3389/fpsyg.2011.00368 22180748 PMC3235774

[B13] Coull J. T. Cheng R. K. Meck W. H. (2011). Neuroanatomical and neurochemical substrates of timing. Neuropsychopharmacology 36, 3–25 10.1038/npp.2010.113 20668434 PMC3055517

[B14] Dehaene S. (1989). The psychophysics of numerical comparison: a reexamination of apparently incompatible data. Percept. Psychophys. 45, 557–566 10.3758/BF03208063 2740196

[B15] Dehaene S. (1992). Varieties of numerical abilities. Cognition 44, 1–42 10.1016/0010-0277(92)90049-N 1511583

[B16] Dehaene S. (2007). Symbols and quantities in parietal cortex: elements of a mathematical theory of number representation and manipulation, in Sensorimotor Foundations of Higher Cognition: Attention and Performance, eds Haggard P. Rossetti Y. Kawato M. (Cambridge, UK: Oxford University Press), 527–574

[B17] Dehaene S. Dupoux E. Mehler J. (1990). Is numerical comparison digital? Analogical and symbolic effects in two-digit number comparison. J. Exp. Psychol. Hum. Percept. Perform. 16, 626–641 10.1037/0096-1523.16.3.626 2144576

[B18] Dehaene S. Izard V. Piazza M. (2005). Control over non-numerical parameters in numerosity experiments. Available online at: www.unicog.org; Last accessed Jun 06, 2013.

[B19] Deloche G. Seron X. (1982a). From one to 1: an analysis of transcoding process by means of neuropsychological data. Cognition 12, 119–149 10.1016/0010-0277(82)90009-9 6890429

[B20] Deloche G. Seron X. (1982b). From three to 3: a differential analysis of skills in transcoding quantities between patients with Broca's and Wernicke's Aphasia. Brain 105, 719–733 10.1093/brain/105.4.719 7139252

[B21] Diamond A. Briand L. Fossella J. Gehlbach L. (2004). Genetic and neurochemical modulation of prefrontal cognitive functions in children. Am. J. Psychiatry 161, 125–132 10.1176/appi.ajp.161.1.125 14702260

[B22] Dickinson D. Elvevåg B. (2009). Genes, cognition and brain through a COMT lens. Neuroscience 164, 72–87 10.1016/j.neuroscience.2009.05.014 19446012 PMC2760675

[B23] Dormal V. Pesenti M. (2012). Processing magnitudes within the parietal cortex, in Horizons in Neuroscience Research, Vol. 8, eds Costa A. Villalba E. (NewYork, NY: Nova Science Publishers), 107–140

[B24] Dumontheil I. Roggeman C. Ziermans T. Peyrard-Janvid M. Matsson H. Kere J. (2011). Influence of the COMT genotype on working memory and brain activity changes during development. Biol. Psychiatry 70, 222–229 10.1016/j.biopsych.2011.02.027 21514925

[B25] Figueiredo V. L. M. (2002). WISC-III: Escala de Inteligência Wechsler para Crianças.Manual Adaptação e Padronização Brasileira. São Paulo: Casa do Psicólogo

[B26] Figueiredo V. L. M. Nascimento E. (2007). Desempenhos nas duas tarefas do subteste dígitos do WISC-III e do WAIS-III. Psicologia Teoria e Pesquisa. 23, 313–318 10.1590/S0102-37722007000300010

[B27] Geary D. C. (1993). Mathematical disabilities: cognitive, neuropsychological, and genetic components. Psychol. Bull. 114, 345–362 10.1037/0033-2909.114.2.345 8416036

[B28] Geary D. C. Hoard M. K. Nugent L. (2012). Journal of Experimental Child Independent contributions of the central executive, intelligence, and in-class attentive behavior to developmental change in the strategies used to solve addition problems. J. Exp. Child Psychol. 113, 1–17 10.1016/j.jecp.2012.03.003 22698947 PMC3392437

[B29] Gibbon J. (1977). Scalar expectancy theory and Weber's law in animal timing. Psychol. Rev. 84, 279 10.1037/0033-295X.84.3.2797844506

[B30] Gilmore C. Attridge N. Inglis M. (2011). Measuring the approximate number system. Q. J. Exp. Psychol. 64, 2099–2109 10.1080/17470218.2011.574710 21846265

[B31] Goldberg T. E. Egan M. F. Gscheidle T. Coppola R. Weickert T. Kolachana B. S. (2003). Executive subprocesses in working memory: relationship to catechol-O-methyltransferase Val158Met genotype and schizophrenia. Arch. Gen. Psychiatry 60, 889 10.1001/archpsyc.60.9.889 12963670

[B32] Halberda J. Mazzocco M. M. Feigenson L. (2008). Individual differences in non-verbal number acuity correlate with maths achievement. Nature 455, 665–668 10.1038/nature07246 18776888

[B33] Hurks P. P. van Loosbroek E. (2012). Time Estimation deficits in childhood mathematics difficulties. J. Learn. Disabil. [Epub ahead of print]. 10.1177/0022219412468161 23263415

[B34] Karoum F. Chrapusta S. J. Egan M. F. (1994). 3-Methoxytryramine is the major metabolite of released dopamine in the rat frontal cortex: reassessment of the effects of antipsychotics on the dynamics of dopamine release and metabolism in the frontal cortex, nucleus accumbens, and striatum by a simple two pool model. J. Neurochem. 63, 972–979 10.1046/j.1471-4159.1994.63030972.x 7914228

[B35] Kessels R. P. C. Van Zandvoort M. J. E. Kapelle L. J. Postma A. De Haan E. H. (2000). The Corsi block-tapping task: standardization and normative data. Appl. Neuropsychol. 7, 252–258 10.1207/S15324826AN0704_8 11296689

[B36] Lopes-Silva J. B. (2013). O papel da Consciência Fonêmica Como Mecanismo Cognitivo Subjacente ao Processamento Numeric. Unpublished Master Dissertation, Universidade Federal de Minas Gerais, Belo Horizonte

[B37] Lustig C. Meck W. H. (2005). Chronic treatment with haloperidol induces working memory deficits in feedback effects of interval timing. Brain Cogn. 58, 9–16 10.1016/j.bandc.2004.09.005 15878723

[B38] Mattay V. S. Goldberg T. E. Fera F. Hariri A. R. Tessitore A. Egan M. F. (2003). Catechol-O-methyltransferase Val158Met genotype and individual variation in the brain response to amphetamine. Proc. Natl. Acad. Sci. U.S.A. 100, 6186–6191 10.1073/pnas.0931309100 12716966 PMC156347

[B39] Mazzocco M. M. M. Feigenson L. Halberda J. (2011). Impaired acuity of the approximate number system underlies mathematical learning disability (dyscalculia). Child Dev. 82, 1224–1237 10.1111/j.1467-8624.2011.01608.x 21679173 PMC4411632

[B40] Meck W. H. (2006). Frontal cortex lesions eliminate the clock speed effect of dopaminergic drugs on interval timing. Brain Res. 1108, 157–167 10.1016/j.brainres.2006.06.046 16844101

[B41] Meck W. H. Church R. M. (1983). A mode-control model of counting and timing processes. J. Exp. Psychol. Anim. Behav. Process. 9, 320–334 10.1037/0097-7403.9.3.320 6886634

[B42] Meck W. H. Church R. M. (1987). Cholinergic modulation of the content of temporal memory. Behav. Neurosci. 101, 457–464 10.1037/0735-7044.101.4.457 2820435

[B43] Mejias S. Mussolin C. Rousselle L. Grégoire J. Noël M. P. (2012). Numerical and nonnumerical estimation in children with and without mathematical learning disabilities. Child Neuropsychol. 18, 550–575 10.1080/09297049.2011.625355 22117818

[B44] Meyer-Lindenberg A. Nichols T. Callicott J. H. Ding J. Kolachana B. Buckholtz J. (2006). Impact of complex genetic variation in COMT on human brain function. Mol. Psychiatry 11, 867–877 10.1038/sj.mp.4001860 16786032

[B45] Mier D. Kirsch P. Meyer-Lindenberg A. (2010). Neural substrates of pleiotropic action of genetic variation in COMT: a meta-analysis. Mol. Psychiatry 15, 918–927 10.1038/mp.2009.36 19417742

[B46] Miller S. A. Dykes D. D. Polesky H. F. (1988). A simple salting out procedure for extracting DNA from human nucleated cells. Nucleic Acids Res. 16, 1215 3344216 10.1093/nar/16.3.1215 PMC334765

[B47] Mills S. Langley K. Van Den Bree M. Street E. Turic D. Owen M. J. (2004). No evidence of association between Catechol-O-Methyltransferase (COMT) Val158Met genotype and performance on neuropsychological tasks in children with ADHD: a case-control study. BMC Psychiatry 4:15 10.1186/1471-244X-4-15 15182372 PMC425584

[B48] Noël M. P. Rousselle L. (2011). Developmental changes in the profiles of dyscalculia: an explanation based on a double exact-and-approximate number representation model. Front. Hum. Neurosci. 5:165 10.3389/fnhum.2011.00165 22203797 PMC3243900

[B49] Piazza M. Dehaene S. (2004). From number neurons to mental arithmetic: the cognitive neuroscience of number sense, in The Cognitive Neurosciences, 3^rd^ Edn., ed Gazzaniga M. (Cambridge, MA: MIT Press), 865–877

[B50] Piazza M. Facoetti A. Trussardi A. N. Berteletti I. Conte S. Lucangeli D. (2010). Developmental trajectory of number acuity reveals a severe impairment in developmental dyscalculia. Cognition 116, 33–41 20381023 10.1016/j.cognition.2010.03.012

[B51] Pinheiro-Chagas P. (2012). In How Many Ways is the Approximate Number System Associated with Mathematics Achievement? The Contributions of Nonsymbolic Comparison, Magnitude Estimation and Approximate Calculation. Unpublished Master Dissertation, Universidade Federal de Minas Gerais, Belo Horizonte

[B52] Prado J. Mutreja R. Zhang H. Mehta R. Desroches A. S. Minas J. E. (2011). Distinct representations of subtraction and multiplication in the neural systems for numerosity and language. Hum. Brain Mapp. 32, 1932–1947 21246667 10.1002/hbm.21159 PMC3117906

[B53] Price G. R. Mazzocco M. M. Ansari D. (2013). Why mental arithmetic counts: brain activation during single digit arithmetic predicts high school math scores. J. Neurosci. 33, 156–163 23283330 10.1523/JNEUROSCI.2936-12.2013 PMC6618631

[B54] Raghubar K. P. Barnes M. A. Hecht S. A. (2010). Working memory and mathematics: a review of developmental, individual difference, and cognitive approaches. Learn. Individ. Dif. 20, 110–122

[B55] Rivera S. M. Reiss A. L. Eckert M. A. Menon V. (2005). Developmental changes in mental arithmetic: evidence for increased functional specialization in the left inferior parietal cortex. Cereb. Cortex 15, 1779–1790 15716474 10.1093/cercor/bhi055

[B56] Rotzer S. Loenneker T. Kucian K. Martin E. Klaver P. Von Aster M. (2009). Dysfunctional neural network of spatial working memory contributes to developmental dyscalculia. Neuropsychologia 47, 2859–2865 19540861 10.1016/j.neuropsychologia.2009.06.009

[B57] Rousselle L. Noël M. P. (2007). Basic numerical skills in children with mathematics learning disabilities: a comparison of symbolic vs. non-symbolic number magnitude processing. Cognition 102, 361–395 16488405 10.1016/j.cognition.2006.01.005

[B58] Ruiz-Sanz J. I. Aurrekoetxea I. Ruiz Del Agua A. Ruiz-Larrea M. B. (2007). Detection of catechol-O-methyltransferase Val158Met polymorphism by a simple one-step tetra-primer amplification refractory mutation system-PCR. Mol. Cell. Probes 21, 202–207 17337160 10.1016/j.mcp.2006.12.001

[B59] Standaert D. Galanter J. M. (2009). Farmacologia da Neurotransmissão Dopaminérgica, in Princípios de Farmacologia: A Base Fisiopatologia da Farmacoterapia, eds Golan D. E. Tashjian A. H. Armstrong E. J. Armstrong A. W. (Rio de Janeiro: Nova Guanabara), 166–185

[B60] Stein D. J. Newman T. K. Savitz J. Ramesar R. (2006). Warriors versus worriers: the role of COMT gene variants. CNS Spectrum 11, 745–748 17008817 10.1017/s1092852900014863

[B61] Tan H. Y. Chen Q. Goldberg T. E. Mattay V. S. Meyer-Lindenberg A. Weinberger D. R. (2007). Catechol-O-methyltransferase Val158Met modulation of prefrontal-parietal-striatal brain systems during arithmetic and temporal transformations in working memory. J. Neurosci. 27, 13393–13401 18057197 10.1523/JNEUROSCI.4041-07.2007 PMC6673107

[B62] Wahlstrom D. White T. Hooper C. J. Vrshek-Schallhorn S. Oetting W. S. Brott M. J. (2007). Variations in the catechol O-methyltransferase polymorphism and prefrontally guided behaviors in adolescents. Biol. Psychiatry 61, 626–632 17014828 10.1016/j.biopsych.2006.05.045

[B63] Walsh V. (2003). A theory of magnitude: common cortical metrics of time, space and quantity. Trends Cogn. Sci. 7, 483–488 14585444 10.1016/j.tics.2003.09.002

[B64] Wardle M. C. de Wit H. Penton-Voak I. Lewis G. Munafò M. R. (2013). Lack of association between COMT and working memory in a population-based cohort of healthy young adults. Neuropsychopharmacology 38, 1253–1263 23337869 10.1038/npp.2013.24 PMC3656369

[B65] Weinshilboum R. M. Otterness D. M. Szumlanski C. L. (1999). Methylation pharmacogenetics: catechol O-methyltransferase, thiopurine methyltransferase, and histamine N-methyltransferase. Annu. Ver. Pharmacol. 39, 19–52 10331075 10.1146/annurev.pharmtox.39.1.19

[B66] Wiener M. Falk W. L. Coslett H. B. (2011). Double dissociation of dopamine genes and timing in humans. J. Cogn. Neurosci. 23, 2811–2821 21261454 10.1162/jocn.2011.21626

[B67] Xu F. Spelke E. S. Goddard S. (2005). Number sense inhuman infants. Dev. Sci. 8, 88–101 15647069 10.1111/j.1467-7687.2005.00395.x

[B68] Zuber J. Pixner S. Moeller K. Nuerk H. C. (2009). On the language specificity of basic number processing: transcoding in a language with inversion and its relation to working memory capacity. J. Exp. Child Psychol. 102, 60–77 18499120 10.1016/j.jecp.2008.04.003

